# A Multi-Step Process of Viral Adaptation to a Mutagenic Nucleoside Analogue by Modulation of Transition Types Leads to Extinction-Escape

**DOI:** 10.1371/journal.ppat.1001072

**Published:** 2010-08-26

**Authors:** Rubén Agudo, Cristina Ferrer-Orta, Armando Arias, Ignacio de la Higuera, Celia Perales, Rosa Pérez-Luque, Nuria Verdaguer, Esteban Domingo

**Affiliations:** 1 Centro de Biologia Molecular “Severo Ochoa” (CSIC-UAM), Cantoblanco, Madrid, Spain; 2 Institut de Biologia Molecular de Barcelona (CSIC), Parc Científic de Barcelona, Barcelona, Spain; 3 Centro de Investigación Biomédica en Red de Enfermedades Hepáticas y Digestivas (CIBERehd), Barcelona, Spain; CNRS, France

## Abstract

Resistance of viruses to mutagenic agents is an important problem for the development of lethal mutagenesis as an antiviral strategy. Previous studies with RNA viruses have documented that resistance to the mutagenic nucleoside analogue ribavirin (1-*β*-D-ribofuranosyl-1-*H*-1,2,4-triazole-3-carboxamide) is mediated by amino acid substitutions in the viral polymerase that either increase the general template copying fidelity of the enzyme or decrease the incorporation of ribavirin into RNA. Here we describe experiments that show that replication of the important picornavirus pathogen foot-and-mouth disease virus (FMDV) in the presence of increasing concentrations of ribavirin results in the sequential incorporation of three amino acid substitutions (M296I, P44S and P169S) in the viral polymerase (3D). The main biological effect of these substitutions is to attenuate the consequences of the mutagenic activity of ribavirin —by avoiding the biased repertoire of transition mutations produced by this purine analogue—and to maintain the replicative fitness of the virus which is able to escape extinction by ribavirin. This is achieved through alteration of the pairing behavior of ribavirin-triphosphate (RTP), as evidenced by *in vitro* polymerization assays with purified mutant 3Ds. Comparison of the three-dimensional structure of wild type and mutant polymerases suggests that the amino acid substitutions alter the position of the template RNA in the entry channel of the enzyme, thereby affecting nucleotide recognition. The results provide evidence of a new mechanism of resistance to a mutagenic nucleoside analogue which allows the virus to maintain a balance among mutation types introduced into progeny genomes during replication under strong mutagenic pressure.

## Author Summary

Viruses that have RNA as genetic material include many important human, animal and plant pathogens. A new strategy against RNA viruses consists in using mutagenic nucleotides. The objective is to provoke an excessive number of mutations, to deteriorate the viral functions to the point that the virus can not survive. One of the mutagens used in research on lethal mutagenesis is ribavirin, extensively employed in clinical practice. Unfortunately, viral mutants that are resistant to ribavirin have been selected, thus facilitating escape from lethal mutagenesis. Here we describe a new mechanism by which foot-and-mouth disease virus (FMDV) can become resistant to ribavirin. Amino acid changes in the viral polymerase, selected by ribavirin, are able to modify the types of mutations produced in the presence of ribavirin. Biochemical data indicate that the alteration of the enzyme changes the pairing behavior of ribavirin, avoiding the production of an excess of some types of mutations, supporting the hypothesis that an unbalanced mutation repertoire is detrimental to the virus. Thus, this new mechanism of resistance to ribavirin is based not as much in limiting the number of mutations in the virus genetic material but in ensuring an equilibrium among different types of mutations that favors viral survival.

## Introduction

The biology of RNA viruses is heavily marked by high mutation rates and quasispecies dynamics, relevant not only for virus evolution but also for viral pathogenesis (review in [1]). The adaptive potential of viral populations as they replicate in the infected hosts represents a formidable problem for the control of viral disease by treatment with antiviral agents. Indeed, selection of viral mutants with decreased sensitivity to one or multiple antiviral inhibitors is an almost systematic occurrence, mainly for riboviruses and retroviruses [2]–[7]. The understanding of pathogenic RNA viruses as quasispecies opened the way to the exploration of a new antiviral approach termed virus entry into error catastrophe or lethal mutagenesis. This strategy was inspired in one of the corollaries of quasispecies theory that asserted that for any replicating system there must be a limit to the average error rate during template copying above which the information conveyed by the system cannot be maintained [8]–[13]. Applied to viruses, this concept implies that an increase of the viral mutation rate by mutagenic agents should result in virus extinction. This prediction has been amply confirmed experimentally with several virus-host systems in cell culture and *in vivo*, using different mutagens, notably nucleoside analogues [14]–[27].

One of the problems for a successful application of lethal mutagenesis to virus extinction is the selection of mutant viruses resistant to mutagenic agents. This problem has been manifested with the selection of picornavirus mutants with decreased sensitivity to the mutagenic base analogue ribavirin (1-*β*-D-ribofuranosyl-1-*H*-1,2,4-triazole-3-carboxamide) (R) [27]–[31]. R is a licensed antiviral agent that has been used over several decades to treat some human viral infections, notably hepatitis C virus (HCV) infections, in combination with interferon (IFN) α or IFN α derivatives [32]–[35]. Since the important discovery that R is mutagenic for poliovirus (PV) [36], R has been used as mutagenic agent in experimental studies of lethal mutagenesis of several RNA viruses [21], [27], [36]–[42]. However, R has several mechanisms of action [43], [44] and whether R mutagenesis participates in the elimination of HCV during treatment of chronic HCV infections is still an open question [45]–[49].

Picornaviruses have contributed to the understanding of the molecular basis of resistance to R. A poliovirus (PV) mutant with decreased sensitivity to R included substitution G64S in its RNA-dependent RNA polymerase (termed 3D). This substitution confers resistance to R through a general increase in template copying fidelity, at the cost of producing mutant spectra of lower complexity than wt PV. Limited mutant spectrum complexity resulted in PV populations which were less adaptable to a complex environment, a direct proof of the essential contribution of high mutation rates to RNA virus adaptability [29], [31]. In the case of foot-and-mouth disease virus (FMDV), resistance to R was associated with substitution M296I in 3D. Contrary to substitution G64S in PV, M296I did not result in increased template-copying fidelity of the 3D of FMDV. Rather, the mutant FMDV restricted the incorporation of RTP into RNA through an alteration of residues in the neighborhood of the active site of 3D that did not have a significant effect on the rate of misincorporation of the standard nucleotides [27], [50], [51].

Replacement M296I was sufficient to prevent extinction of FMDV by high concentrations of R, but the virus was extinguished by an alternative mutagenic treatment that included 5-fluorouracil (FU) [52]. Since R-resistance mutations can jeopardize viral extinction by lethal mutagenesis, it is of upmost importance to understand the molecular mechanisms of R-resistance, with the objective of designing adequate protocols for virus extinction. FMDV with replacement M296I was selected upon passage of the virus in the presence of increasing R concentrations in the range of 200 µM to 800 µM included in the cell culture medium [27]. Since R reduces the viability for BHK-21 cells in 40% after two days of treatment [42], [52], [53] (see Materials and Methods), and allowed virus replication, we tested the response of FMDV to replication in the presence of high concentration of R. Here we report that FMDV populations replicated in the presence of concentrations of R in the range of 800 µM to 5000 µM, accumulated two additional amino acid substitutions in 3D in a step-wise fashion. The substituted polymerase displays a new molecular mechanism of R-resistance based on modulation of the types of R-induced misincorporations during RNA synthesis, based on an alteration of the pairing preference of R opposite C and U. In this manner, the mutant FMDV, but not the wild type FMDV, produces progeny RNA that shows a balanced distribution of transition types despite replicating in the presence of R. Studies of polymerization activity by the purified polymerases suggest that a single amino acid substitution in a loop of the fingers domain is the alteration chiefly responsible of the altered mutational pattern. The crystal structures of the substituted polymerases in complex with RNA show a conformational change in the template entry channel of the polymerase, that may affect the binding of the ssRNA template to 3D, mainly at the base of the template which is immediately downstream of the position that receives the incoming nucleotide. Alteration of the position of the template RNA at the active site of the enzyme may affect nucleotide recognition and modify the transition mutation pattern in the presence of R. The findings establish a new mechanism of lethal mutagenesis-escape in viruses which rests on regulation of the mutational spectrum in progeny viral genomes.

## Results

### Adaptation of FMDV to high ribavirin concentrations

**Figure 1:**
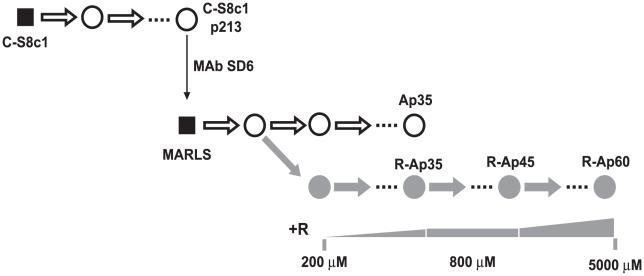
Passage history of FMDV in the presence of increasing cencentrations of ribavirin. Biological clone C-S8c1of FMDV was subjected to up to 460 serial passages in BHK-21 cells [70], [92]. Al passage 213, biological clone MARLS was selected by its resistance to neutralization by monoclonal antibody (MAb) SD6 [55], and the population derived from the clone was subjected to serial passages either in the absence (white circles) or presence (grey circles) of increasing concentrations of ribavirin (R) [60]. In this scheme biological clones (virus derived from a single plaque developed on a BHK-21 cell monolayer) are indicated as black squares, and uncloned populations as circles; “p” indicates passage number. The concentrations of R included in the culture medium are indicated below the corresponding passages. The procedures involved in the isolation of the initial FMDV C-S8c1 clone and in infections of BHK-21 cells have been described in our previous studies [55], [60], [70], [92] and are detailed in Materials and Methods.

A biological clone of FMDV termed C-S8c1 is the standard virus used in our studies of molecular evolution and lethal mutagenesis of FMDV [54]. FMDV C-S8c1 was serially passaged in BHK-21 cells, and a monoclonal antibody (MAb)-escape mutant termed MARLS was isolated from the population at passage 213 [55]. FMDV MARLS was then subjected to passages in the presence of 200 µM to 800 µM R in the culture medium, resulting in selection of population R-Ap35 which included amino acid substitution M296I in 3D [27] (Figure 1). Population R-Ap35 displayed higher fitness than wild type FMDV in the presence of R but not in its absence [27]. Although M296I was the only replacement that became dominant in FMDV populations passaged in the presence of R (the diagnostic nucleotide band in the consensus sequence did not indicate any detectable amount of an alternative nucleotide), other substitutions in 3D that did not reach dominance were also observed [56]. To study the response of FMDV to replication in the presence of higher concentrations of R, population R-Ap35 was subjected to 10 additional passages in the presence of 800 µM R, and then to 15 passages in the presence of increasing concentration of R (from 1000 to 5000 µM), to obtain population R-Ap60 (Figure 1). Populations R-Ap60 and R-Ap35 displayed a similar mutation frequency in their mutant spectra (Table 1), but the specific infectivity [plaque-forming-units (PFU)/amount of viral RNA] of R-Ap60 was 10-fold lower than that of R-Ap35 (Table 1). These results suggest that virus replication under increased R concentrations led to loss of virus viability, not necessarily correlated with a significant increase of average mutation frequency.

**Table 1:** Specific infectivity of mutant FMDV populations.

Population	Mutation Frequency^a^	Virus titer (PFU/ml)^b^	RNA (molecules/ml)^b^	Specific infectivity^b^
R-Ap35	4.2×10^−3^	1.4±0.1×10^7^	0.8±0.1×10^11^	18×10^−5^
R-Ap60	3.2×10^−3^	0.2±0.0×10^7^	1.0±0.0×10^11^	2×10^−5^

a Mutation frequencies are based on the analysis of 18,620 nucleotides of the mutant spectrum of R-Ap35 (14 molecular clones) and 19,950 nucleotides for R-Ap60 (15 molecular clones). For each clone the genomic region analysed comprised residues 6610 to 8020 (residue numbering is according to [93]).

b Values of virus titer and RNA molecules are the average of three determinations, and standard deviations are given. The specific infectivity was calculated as PFU/RNA molecules (ratio of values in the third to the fourth column). Procedures are detailed in Materials and Methods.

To test whether population of R-Ap60 was better adapted to R than population R-Ap35, the relative fitness of the two populations was determined in growth-competition experiments in the presence and absence of R, using as reference the virus population Ap35 (which is FMDV MARLS passaged 35 times in the absence of R, as described in Materials and Methods and in [27]). The results (Table 2) indicate that R-Ap60 is better adapted than R-Ap35 to replicate in the presence of R. The adaptation of R-Ap60 resulted from a specific response of FMDV to R, since the fitness of R-Ap60 relative to R-Ap35 in the presence of FU and guanidine hydrochloride (GuH) (an alternative mutagenic combination used in lethal mutagenesis of FMDV [52]) was 0.7 (Table 2). Thus, FMDV underwent a progressive adaptation to replicate efficiently under high R concentration.

**Table 2:** Fitness of mutant FMDV populations.

Population	Fitness Value^a^			
	Absence of R	800 µM R	5000 µM R	FU (300 µg/ml) + GuH (4 mM)
R-Ap35	0.5R^2^ = 0.87	4R^2^ = 0.95	n.d.	n.d.
R-Ap60	0.6R^2^ = 0.75	11R^2^ = 0.98	15R^2^ = 0.99	0.7R^2^ = 0.82

a Relative fitness values were determined by growth competition experiments between Ap35 (reference virus) and either population R-Ap35 or R-Ap60 in the absence or presence of the indicated drug concentrations (R, ribavirin; FU, 5-fluorouracil; GuH, guanidine hydrochloride). n.d., not determined. R^2^ values of the fitness vector (linear regression) are given the corresponding box. Procedures are detailed in Materials and Methods.

### Adaptation of FMDV to high ribavirin concentrations entails additional substitutions in 3D

To study whether adaptation of FMDV to increased concentrations of R was associated with additional substitutions in 3D, the consensus nucleotide sequence of the 3D-coding region of R-Ap35 and R-Ap60 was analyzed (Table 3). Two new mutations were found as dominant in R-Ap60: C6739U (that gives rise to amino acid substitution P44S in 3D), and C7114U (that gives rise to P169S in 3D). In addition, 3D maintained as dominant substitution M296I which was already dominant in R-Ap35 [27], [50]. P44S but not P169S was detected in a 70% proportion in R-Ap35, as evidenced by analysis of both the consensus sequences and their corresponding mutant spectra (Table 3). These results suggest that the three substitutions in 3D were selected sequentially during replication in the presence of increasing concentrations of R: first M296I, then P44S and finally P169S.

**Table 3:** Consensus sequence and mutant spectrum composition of the 3D-coding region of FMDV R-Ap35 and R-Ap60, and deduced amino acid substitutions.

Mutations in the consensus sequence^a^		Amino acid substitution^b^	Frequency of amino acid substitutions in mutant spectra^c^	
R-Ap35	R-Ap60		R-Ap35	R-Ap60
C6739C/U	C6739U	P44S	**0.71**	**1**
	A6741G	=		
	C6744U	=		
	C6975U	=		
A7003A/U		I132V	**0.50**	**0.13**
G7098G/A	G7098A	=		
	C7114U	P169S	**0**	**1**
C7350C/U		=		
	C7374A	=		
C7404C/U	C7404U	=		
	C7413U	=		
	U7419C	=		
G7453G/A		E282K	**0.43**	**0**
G7497A	G7497A	M296I	**1**	**1**
C7506C/A	C7506A	=		
C7548C/U	C7548U	=		
	G7554U	=		
	U7569C	=		
C7935C/U	C7935U	=		
	C7947U	=		

a Mutations found in the consensus sequence at the 3D-coding region of populations R-Ap35 and R-Ap60, relative to the genomic sequence of the parental FMDV MARLS (Figure 1). Two nucleotides separated by a dash indicate the presence of a mixture, as judged from the nucleotide sequence chromatogram. FMDV genomic residues are numbered as previously described [93]. Procedures for nucleotide sequencing are described in Materials and Methods.

b Amino acid substitutions in 3D that result from the mutations found in R-Ap35 or R-Ap60 populations indicated in the first two columns. Replacements that are totally imposed in R-Ap35 or R-Ap60 are underlined. “ = ”means a synonymous mutation.

c Frequency of the amino acid that results form each mutation, as indicated in the third column (S44, V132, S169, K282, I296) found in two or more molecular clones from the corresponding mutant spectrum. Mutations and deduced amino acid substitutions are based on the analysis of 18,620 nucleotides of the mutant spectrum of the 3D-coding region from population R-Ap35 (14 clones), and 19,950 nucleotides in the case of R-Ap60 (15 clones). Procedures for mutant spectrum analysis are described in Materials and Methods.

### The replacements in 3D increase FMDV fitness in the presence of ribavirin

**Figure 2:**
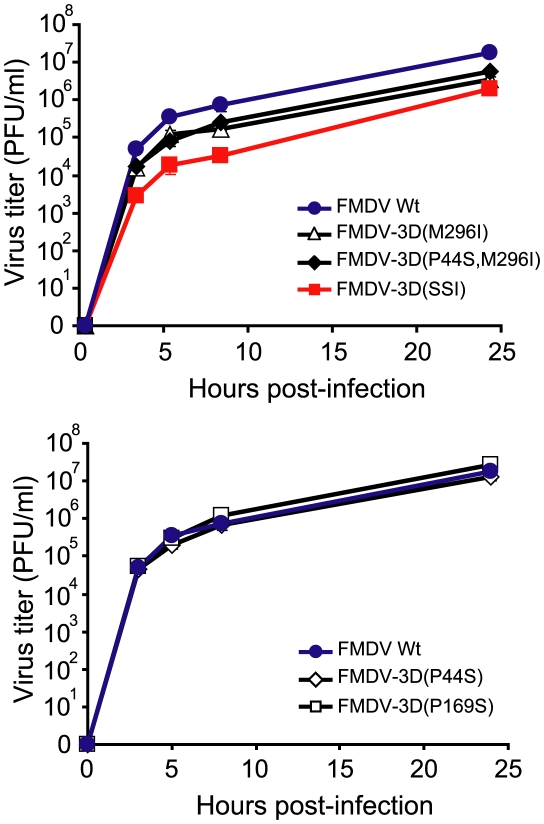
Progeny production in BHK-21 cells infected with viruses encoding mutant polymerases. Kinetics of progeny production of infectious virus in BHK-21 infected cells infected at a MOI of 0.5 PFU/cell by the indicated FMDVs. Data have been divided in two separate graphs for clarity. Results are the average of three determinations and standard deviations are given. Procedures are described in Materials and Methods.

To investigate the effect of the 3D substitutions in the sequence context of pMT28 (the plasmid from which C-S8c1 is expressed [57]) without possible confounding effects of other mutations in the viral genome, plasmids pMT28-3D(M296I), pMT28-3D(P44S), pMT28-3D(P169S), pMT28-3D(P44S, M296I) and pMT28-3D(SSI) (SSI means the presence of the triple replacement P44S, P169S and M296I in 3D) were constructed as described in Materials and Methods. These plasmids encode the genome of C-S8c1 with the mutations that give rise to the indicated substitutions in 3D, as the only difference with respect to the wild type sequence (pMT28 or C-S8c1 [57]). BHK-21 cells were transfected with the corresponding RNA transcripts and the rescued viruses [termed FMDV 3D(M296I), FMDV 3D(P44S), FMDV 3D(P169S), FMDV 3D(P44S, M296I) and FMDV 3D(SSI), respectively] were tested regarding infectious progeny production (Figure 2). FMDV 3D(M296I), FMDV 3D(P44S, M296I), and FMDV 3D(SSI), but not FMDV 3D(P44S) and FMDV 3D(P169S), showed lower progeny production in the absence of R. Fitness measurements in the absence and presence of R (Table 4) indicate that the triple replacement P44S, P169S and M296I conferred on the virus a selective advantage in the presence of R. The addition of P169S to a virus harboring P44S and M296I provided an advantage during replication in the presence of 5000 µM but not 800 µM R. A direct competition showed a selective advantage of FMDV(SSI) over FMDV 3D(M296I) in the presence of 5000 µM R. P44S and P169S individually did not inflict a fitness cost upon the virus in the absence of R, whereas M296I and the triple combination did (Table 4).

**Table 4:** Fitness value of mutant FMDV populations.

FMDV in the competition	Fitness Value^a^		
	Absence of R	800 µM R	5000 µM R
**P44S**/Wt	1.1R^2^ = 0.93	1.2R^2^ = 0.62	n.d.
**P169S**/Wt	1.2R^2^ = 0.90	2R^2^ = 1.00	3R^2^ = 0.96
**M296I**/Wt	0.6R^2^ = 0.83	4R^2^ = 0.97	n.d.
**SSI**/Wt	0.7R^2^ = 0.91	4R^2^ = 1.00	2R^2^ = 0.93
**SSI**/(M296I)	0.5R^2^ = 0.96	0.6R^2^ = 0.73	6R^2^ = 0.93
**SSI**/(P44S, M296I)	0.2R^2^ = 0.92	0.3R^2^ = 0.95	2R^2^ = 0.80

a Relative fitness values obtained in the competitions between the clonal FMDV population depicted in bold letters (that indicate the amino acid substitution in 3D; SSI means the triple mutant FMDV 3D(P44S, P169S, M296I) relative to the population depicted in regular letters [wild type, wt, mutant FMDV (M296I) or the double mutant FMDV 3D(P44S, M296I)]. Fitness was determined in the absence or in the presence of the indicated concentration of R. n.d., not determined. R^2^ values of the fitness vector (linear regression) are given in each case. Procedures are detailed in Materials and Methods.

The three dominant substitutions in the polymerase of the clonal FMDV 3D(SSI) failed to reproduce the fitness difference between populations R-Ap60 and R-Ap35 in the presence of R (compare Tables 2 and 4). This means that factors other than the three dominant replacement in 3D must intervene to confer the growth advantage of R-Ap60 in the presence of R (see Discussion). These additional factors are presently under investigation.

### The mutant spectrum of pMT28-3D(SSI) passaged in the presence of ribavirin reveals an unexpected repertoire of mutations

The mutant spectra of FMDV populations passaged in the absence of drugs display a balance among the four types of transition mutations with a slight dominance of U→C and A→G versus C→U and G→A [27], [58]–[60]. However, FMDV replication in the presence of R inverted this trend, and resulted in a clear dominance of C→U and G→A transitions [27], [42], as also observed with poliovirus (PV) replicating in the presence of R [21], [36]. It was suggested that the bias in favor of C→U and G→A observed in FMDV could reflect a preference for ribavirin-5′-monophosphate (RMP) to be incorporated by 3D polymerase more efficiently opposite to C than U in the template, but this was not supported by the biochemical data on the incorporation of RMP by purified FMDV 3D using heteropolymeric template-primers [27]. The biased mutation types during intracellular viral replication could be influenced by the decrease in intracellular GTP levels due to the inhibition of inosine monophosphate dehydrogenase (IMPDH) by ribavirin-monophosphate (RMP) [42], [43], [61], although previous studies suggested a minor effect of decreased intracellular concentration of GTP on the mutagenic activity of R on FMDV [42].

To explore possible variations in mutation frequency and in the types of mutations as a result of R treatment, FMDV wild type (Wt) (rescued from plasmid pMT28) and FMDV 3D(SSI) were subjected either to five passages in the absence of R (that gave rise to populations abbreviated as Wt-5 and SSI-5, respectively) or to four passages in the presence of 5000 µM R (that gave rise to populations abbreviated as R-Wt-4 and R-SSI-4, respectively). The comparison of mutation frequencies in the mutant spectrum of the different populations showed a 3.5-fold increase in both viruses after passage in the presence of R, as expected [27], [42], [52], but no significant difference in mutation frequency between Wt-5 and SSI-5 (t = 0.45, P>0.1; t Student's test) or between R-Wt-4 and R-SSI-4 (t = 1.16, P>0.1; t Student's test) was seen (Table 5). The mutant spectra of Wt-5 and SSI-5 showed a similar distribution of mutation types (χ^2^ = 0.02, P>0.1; χ^2^ test), with a slight dominance of U→C and A→G, as previously found in FMDV populations that had replicated in the absence of R [27], [58]–[60]. However, the mutation pattern of R-SSI-4 was unexpected for a virus passaged in the presence of a high concentration of R. While in the mutant spectrum of R-Wt-4 the bias in favor of C→U and G→A transitions reached 80%, in R-SSI-4 these transition types amounted to 34% of the total number of mutations. Thus, the repertoire of transition types remained balanced in FMDV 3D(SSI) despite replication in the presence of R, in sharp contrast with FMDV Wt which presented a gross imbalance in favor of C→U and G→A transitions. The ratio (C→U)+(G→A)/(U→C)+(A→G) in the mutant spectra of R-SSI-4 and SSI-5 was virtually identical (χ^2^ = 0.49, P>0.1) (Table 5). This result indicates a remarkable insensitivity to the presence of R regarding the mutations represented in progeny RNA when replacements P44S, P169S and M296I were present in 3D. The insensitivity to R could not be attributed to the absence of replication of FMDV 3D(SSI) in the presence of R since in fact this virus replicates more efficiently than wild type in the presence of R (Table 4). Thus, R-Wt-4 and R-SSI-4 displayed a highly significant difference regarding mutation types in their mutant spectra (χ^2^ = 13.3, P<0.001). These results suggest that adaptation of FMDV to high R concentrations was related to modulation of the types of transitions imposed by the pairing behavior of RMP, preventing a highly biased mutation pattern in progeny genomes.

**Table 5:** Frequency and types of transition mutations in the mutant spectra of FMDV populations passaged in the presence or absence of 5000 µM R^a^.

FMDV population	Mutation frequency (substitutions/nucleotide)	(C→U)+(G→A) (% of total)^b^	(U→C)+(A→G) (% of total)^b^	Ratio 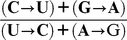
**Wt-5**	6×10^−4^	22	78	0.28
**SSI-5**	4×10^−4^	27	55	0.49
**R-Wt-4**	19×10^−4^	80	20	4.00
**R-SSI-4**	13×10^−4^	34	63	0.53

a Mutation frequencies and mutation types are based on the analysis of 31,770 nucleotides of the mutant spectrum of Wt-5 (23 molecular clones), 23,780 nucleotides for SSI-5 (17 molecular clones), 32,664 nucleotides for R-Wt-4 (24 molecular clones) and 26,332 nucleotides for R-SSI-4 (19 molecular clones). For each clone the genomic region analysed comprised residues 6610 to 8020.

b Percentage of C→U plus G→A transitions or U→C plus A→G transitions relative to the total number of mutations found in the mutant spectra of the indicated populations.

### FMDV 3D(SSI) is resistant to extinction by ribavirin

**Figure 3:**
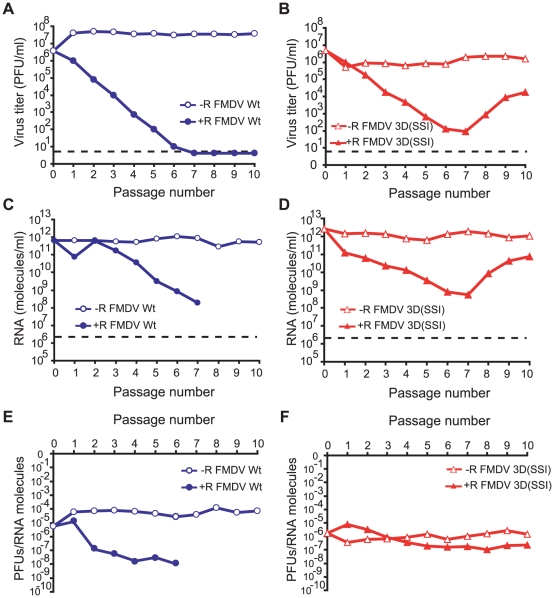
Effect of ribavirin on progeny infectivity and viral RNA in serial infections with FMDV Wt or FMDV 3D(SSI). (A) BHK-21 cells (2×10^6^) were infected with wild type FMDV (Wt) (progeny of infectious clone pMT28 [57], [70]) at a multiplicity of infection of 0.3 PFU/cell. In successive passages, the same number of cells was infected with 1∶10 of the volume of the supernatant from the previous passage in the absence (−R) or in the presence of 5000 µM R (+R). The discontinuous line indicates the limit of detection of viral infectivity. (B) Same as (A), using mutant FMDV 3D(SSI). (C) and (D) FMDV RNA levels in the supernatants of BHK-21 cells infected with FMDV Wt or FMDV 3D(SSI) in the absence (−R) or presence (+R) of 5000 µM R. The discontinuous line indicates the limit of detection of viral RNA. (E) and (F) Specific infectivity (PFUs/RNA molecules) calculated from the data in panels (A) to (D). The specific infectivity from passage 7 of FMDV Wt in the presence of R was not calculated due to undetectable virus titer (<5 PFU/ml). Procedures are described in Materials and Methods.

To investigate whether FMDV 3D(SSI) was resistant to extinction by R, FMDV Wt and FMDV 3D(SSI) were subjected to serial cytolytic passages in BHK-21 cells in the presence or absence of 5000 µM R. The wild type population was extinguished by passage 7, as expected [52] (Figure 3A). In contrast, FMDV 3D(SSI) was not extinguished and, interestingly, the virus titer decreased until passage 6, and then it increased (Figure. 3B). While the specific infectivity of FMDV Wt decreased in the presence of R, the specific infectivity of FMDV 3D(SSI) was very similar in the R-treated and untreated populations (Figure. 3C,D). The consensus nucleotide sequence of the genome of FMDV 3D(SSI) at passage 10 in the presence of R indicated that only one additional mutation (in the non-structural protein 2C-coding region) became dominant in the entire genome (data not shown). The biological significance of this mutation in the 2C-coding region is under investigation. Substitutions P44S, P169S and M296I in 3D were maintained as dominant in the population that escaped extinction and gained replication capacity.

To further substantiate the hypothesis that substitutions P44S, P169S and M296I confer a selective advantage in the presence of R but not in the presence of another mutagen that induces a different mutational repertoire, growth-competition experiments between FMDV Wt and FMDV 3D(SSI) were carried out in the presence of either R or FU (a mutagen which induces mainly U→C and A→G transition in FMDV [58], [60]) or a mixture of R and FU. The results (Table 6) show that a selective advantage of FMDV 3D(SSI) was manifested in the competitions carried out in the presence of R, but not in the presence of FU or of a mixture of R and FU.

**Table 6:** Fitness of FMDV 3D(SSI) relative to FMDV Wt in the presence of R, FU, or a mixture of R and FU.

Drug	Fitness value SSI/Wt^a^
**Absence of drug**	0.9±0.1R^2^ = 0.64; R^2^ = 0.07; R^2^ = 0.44
**Ribavirin**	2.9±0.3R^2^ = 1.00; R^2^ = 0.99; R^2^ = 0.98
**5-Fluorouracil**	1.0±0.1R^2^ = 0.99; R^2^ = 0.69; R^2^ = 0.03
**Ribavirin + 5-Fluorouracil**	1.4±0.5R^2^ = 0.97; R^2^ = 0.98; R^2^ = 0.27

a Growth-competition experiments were initiated by infecting 2×10^6^ BHK-21 cells with 7×10^5^ PFU of FMDV Wt and FMDV 3D(SSI) in the absence of drugs or in the presence either of 5000 µM R, 2000 µM FU or a mixture of 5000 µM R and 2000 µM FU, as indicated in the first column. Progeny virus was used to infect fresh BHK-21 cells monolayers under the same conditions. A total of 3 passages were carried out. The initial population and the populations at passages 1, 2 and 3 were sequenced and the proportion of the two competing genomes were determined by nucleotide sequencing and measurement of the areas of the relevant bands. The three R^2^ values given correspond to each of the three determinations. The average fitness value and standard deviation are given. Drug treatment of cells and calculation of relative fitness are described in Materials and Methods.

### Substitution P44S decreases 3D activity

To investigate the effects of substitutions P44S, P169S and M296I on 3D activity, the wild type polymerase (termed 3DWt), the polymerases that include the individual substitutions [termed 3D(P44S), 3D(P169S) and 3D(M296I)] and the polymerase with the three substitutions [termed 3D(SSI)] were purified as detailed in Materials and Methods and compared in RNA polymerization, VPg-uridylylation and RNA-binding assays (Table 7). 3D(P44S) and 3D(SSI) showed lower activity than the other enzymes in poly(rU) synthesis and in binding to heteropolymeric RNA. In addition, 3D(P44S) displayed a modest decrease in VPg-uridylylation activity. The comparison of activity values *in vitro* suggests that amino acid P44S inflicted a cost upon 3D function.

**Table 7:** Activity of mutant FMDV polymerases (3D)^a^.

3D	Poly(rU) synthesis^b^ (pmol UTP µg^−1^ min^−1^)	VPg uridylylation^c^ (pmol UTP µg^−1^ min^−1^)	RNA binding^d^ (% RNA retarded)
**wt**	161±9	0.43±0.02	62±11
**P44S**	89±18	0.26±0.07	33±11
**P169S**	182±29	0.41±0.06	58±12
**M296I**	182±20	0.49±0.04	63±8
**SSI**	104±15	0.46±0.10	34±10

a Procedures for the expression and purification of wild type and mutant 3Ds and the assays used have been previously described [27], [50], [63], [65], [67], and are detailed in Materials and Methods.

b Poly(rU) synthesis is calculated as pmol UTP incorporated per µg of enzyme per min.

c VPg uridylylation activity is calculated as pmol UTP incorporated into VPg per µg of enzyme per min.

d RNA binding is calculated as % of RNA template-primer retarded by 1.8 µM enzyme at 10 min reaction time, by dividing the labelled bound (retarded) product by the total labelled RNA and multiplying by 100. The relative amount of labeled RNA was visualized and quantitated with a Phosphorimager (BAS-1500; Fuji).

### Mutant polymerase 3D(SSI) is deficient in the incorporation of ribavirin-5′-triphosphate and shows a bias to favor misincorporation of RMP opposite U

**Figure 4:**
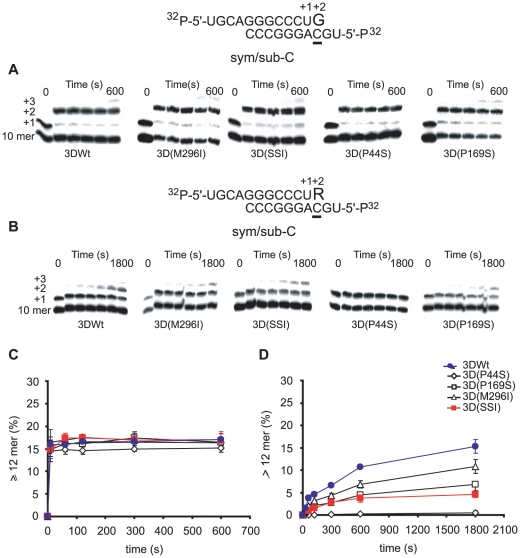
Incorporation of nucleotides into sym/sub-AC by mutant FMDV polymerases. (A) Kinetics of incorporation of GMP into sym/sub-AC (sequence shown at the top) by the indicated FMDV polymerases. The reactions were initiated by addition of 50 µM GTP after the formation of 3D-RNA(n+1) complex, as described in Materials and Methods. At different time points the reaction was quenched by addition of EDTA. (B) Same as (A), except that the reaction was started by addition of 50 µM RTP after the formation of the 3D-RNA (n+1) complex. (C) Percentage of primer elongated to position +2 (12 mer or larger RNAs synthesized), calculated from the densitometric analysis of the electrophoreses shown in A. The results are the average of three independent experiments, and standard deviations are given. (D) Same as (C) for the incorporation of RMP at position +2 (12 mer) calculated from the densitometric analysis of the electrophoreses shown in (B). Procedures are detailed in Materials and Methods.

**Figure 5:**
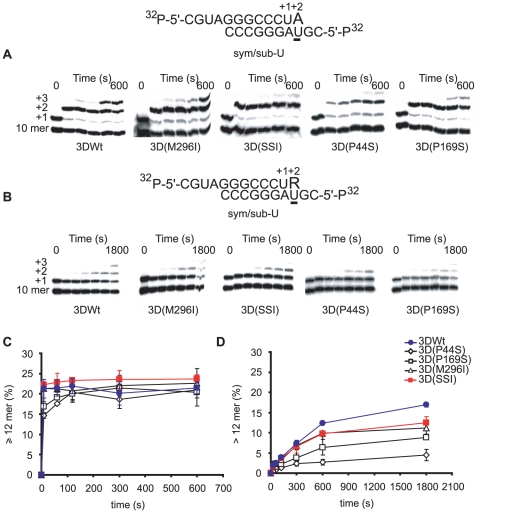
Incorporation of nucleotides into sym/sub-AC by mutant FMDV polymerases. Kinetics of incorporation of AMP into sym/sub-AU (sequence shown at the top) by the indicated polymerases. The reactions were initiated by addition of 50 µM ATP after the formation of 3D-RNA (n+1) complex. At different time points the reaction was quenched by addition of EDTA. (B) Same as (A), except that the reaction was started by addition of 50 µM RTP after the formation of 3D-RNA (n+1) after formation of the 3D-RNA (n+1) complex. (C) Percentage of primer elongated (12 mer or larger RNAs synthesized) calculated from the densitometric analysis of the electrophoreses shown in (A). The results are the average of three independent experiments, and standard deviations are given. (D) Same as (C) for the incorporation of RMP at position +2 (12 mer) calculated from the densitometric analysis of the electrophoreses shown in (B). Procedures are detailed in Materials and Methods.

Previous studies documented that 3D(M296I) displayed a defect in the incorporation of RTP opposite either U and C, in comparison with 3D Wt [27], [50]. The capacity of 3DWt, 3D(P44S), 3D(P169S), 3D(M296I) and 3D(SSI) to use RTP as substrate, and to incorporate RMP opposite U and C was investigated using two symmetrical/subtrate template-primer RNAs [62], termed sym/sub-AC and sym/sub-AU (AC and AU indicate the two template residues that direct the elongation of the primer RNA in two positions, and that allow quantification of the incorporation of R at position +2, opposite C and U, respectively) (Figures 4 and 5). No significant differences in the incorporation GTP of and ATP by 3DWt and 3D(SSI) were observed. Additional experiments were carried out using 1 µM GTP or ATP at 37°C or 33°C, with sym/sub-AC, sym/sub-AU, sym/sub-C and sym/sub-U; again, no differences in the incorporation by 3DWt and 3D(SSI) were observed (Supplementary material, Figures S1, S2, S3). In all cases, the mutant 3Ds were less efficient in RMP incorporation than 3DWt. Interestingly, the incorporation of RMP opposite C was 3-fold lower for 3D(SSI) than for 3D(M296I), but no such difference was observed when the incorporation of RMP was measured opposite U (Figure 5). 3D(P44S) displayed undetectable incorporation of RMP opposite C in the template (<0.5% of elongated sym/sub-AC), and only a modest incorporation opposite U (5±1% of elongated sym/sub-AU). Thus, the incorporation of RMP by 3D(P44S) is at least 10-fold more efficient opposite U than opposite C, suggesting that P44S is the substitution responsible for the biased repertoire of transition mutations during replication of FMDV 3D(SSI) in the presence of R. Comparison of the results of 3D activity (Table 7) and of RMP incorporation (Figures 4 and 5) suggests that substitutions P169S and M296I could exert some compensatory effect to confer 3D with P44S a sufficient polymerization activity while maintaining a limited and biased RMP incorporation. The specific bias displayed by 3D(SSI) against incorporation of RMP opposite C determined *in vitro*, is consistent with the proportion of transition types observed during replication of FMDV 3D(SSI) in the presence of R during infections of BHK-21 cells (compare Figures 4, 5 and Table 5). FMDV 3D(SSI) populations did not display a significantly lower mutant spectrum complexity than FMDV Wt (Table 5), suggesting that the biased incorporation of R is not directly linked to a significant change in the average template copying fidelity as regards the misincorporation of standard nucleotides. However, this point is under further investigation.

### The structure of the 3D mutant polymerases and their complexes with RNA

To identify possible structural modifications of the viral polymerase associated with the important alterations of the mutational spectrum in progeny RNA, and to investigate how these modifications can affect RNA binding and polymerase activity, the different mutant 3Ds were incubated with the heteropolymeric sym/sub-U RNA of sequence 5′GCAUGGGCCC3′, crystallized, and analyzed by X-ray diffraction. Sym/sub- U indicates that U is the template residue which directs the incorporation of A to produce a +1 elongation product. This is the same RNA used in our previous structural studies with FMDV 3D [63]–[65] (see Materials and Methods). For the structural comparisons RNA residues are numbered starting at the 5′ terminal nucleotide.

Two different crystal forms were obtained (Table 8); the single mutants 3D(P44S) and 3D(P169S) incubated with sym/sub-U RNA crystallized in the tetragonal P4_2_2_1_2 space group. The RNA molecule appeared mostly disordered in the two structures. In contrast, 3D(SSI) crystallized in the trigonal P3_2_21 space group with the sym/sub-U RNA incorporated in the structure. Since the biochemical results indicate that P44S plays a critical role in the misincorporation of RMP into RNA by 3D, and a 3D(P44S)-RNA complex was not obtained, we attempted the crystallization of the double mutant 3D(P44S, M296I) in complex with RNA. 3D(P44S, M296I) also crystallized in the space group P3_2_21 space group, with the sym/sub-U RNA incorporated in the structure.

**Table 8:** Data collection and refinement statistics^a^.

	3D(P44S)	3D(P169S)	3D (P44S,M296I)	3D(SSI)
**Resolution (Å)**	30- 2.28	30- 2.6	30- 2.6	30- 2.5
**Space group**	P4_1_2_1_2	P4_1_2_1_2	P3_2_21	P3_2_21
**Unit cell dimensions (Å)**	a = b = 93.53c = 121.010	a = b = 93.834c = 121.687	a = b = 93.713c = 99.723	a = b = 93.836c = 99.867
**Total data**	160380	110453	83589	129958
**Unique data**	24188	14688	15946	18935
**Completeness(%)**	96.6	99.8	99.6	99.6
**Mean I/σ (Di)**	16.6	18.4	14.8	16.2
**Rmerge (%)**	7.6	10.3	11.0	9.3
**R_work_**	23.4	22.5	22.6	22.2
**R_free_**	26.5	26.0	27.4	25.4
**Number of residues**				
**Protein**	476	476	476	476
**Solvent atoms**	-	45	44	41
**Ions**	-	2	2	0
**Ribonucleotides**	-	5	12	12
**R.m.s. deviation from ideal geometry**				
**Bond lengths (Å)**	0.006	0.006	0.006	0.006
**Bond angles (°)**	0.85	0.84	0.86	0.84
**Avg. Temp. Factors (Å)**				
**Protein**	31.5	34.0	26.0	27.7
**Solvent and ions**	23.9	26.3	31.179	33.6
**Ribonucleotides**	-	-	69.0	66.2

a Crystallographic procedures are described in Materials and Methods.

**Figure 6:**
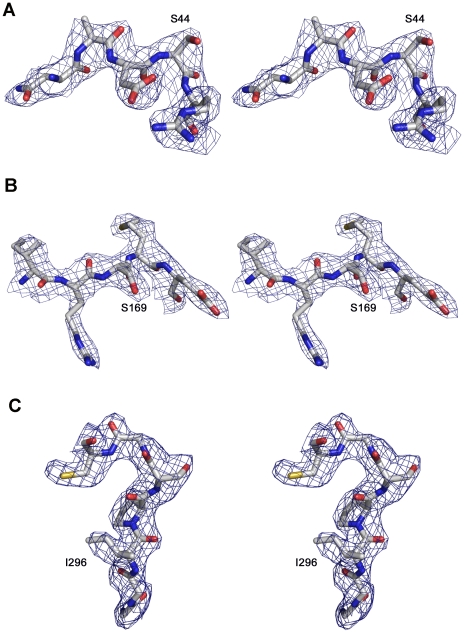
FMDV 3D residues around the substituted sites. Stereoviews of σ_A_-weighted |F_o_|-|F_c_| electron density maps at 2.5 Å resolution (contoured at 3 σ) around the mutated amino acids (A) S44, (B) S169 and (C) I296. The substituted residues and surrounding amino acids were omitted from the phasing model. The model is placed inside in ball and stick representation and colored in atom type code. The names of the mutated residues are labeled.

Further attempts to obtain the structures of ternary complexes, using ATP or RTP were unsuccessful, despite using different substrate concentrations and incubation times. The X-ray structures were determined to 2.2 Å and 2.6 Å resolution for 3D(P44S) and 3D(P169S), respectively, and to 2.6 Å and 2.5 Å for 3D(P44S, M296I) and 3D(SSI), respectively (Table 8). The quality of the resulting difference electron density maps allowed the unequivocal tracing of the mutated and surrounding residues that were omitted from the initial models to eliminate model bias (Figure 6). The analysis of the electron density showed also the presence of the duplex portion of the template-primer RNA in the central channel of the polymerase of the trigonal 3D(P44S, M296I) and 3D(SSI) crystals. In addition, two of the four nucleotides of the 5′ overhang moiety (A3 and U4) were reasonably well defined, occupying the template channel, in both structures.

No major structural changes were observed in the polymerase active site when the structures of the different polymerases (either unbound or bound to RNA) were compared. The structural superimpositions of all 476 amino acids residues of 3D(P44S) and 3D(P169S) and of 3D(P44S, M296I) onto the 3D(SSI) showed root mean square deviation (rmsd) values of 0.46Å, 0.35Å and 0.22Å, respectively.

Subtle domain movements, in particular a ∼1° rotation of the thumb domains relative to the fingers, were observed between the unbound, tetragonal, and the RNA-bound, trigonal structures when the individual domains were superimposed. When the unbound and RNA-bound structures were compared for 3DWt a similar small rotation (∼2°) was also observed. As a consequence of this rotation, the active site appeared more closed in the unbound state. Thus, the changes observed seem to be a consequence of either RNA-binding, or of the different packing constraints in the tetragonal and trigonal space groups or both, but they do not seem to be related to the presence of substitutions P44S or P169S.

**Figure 7:**
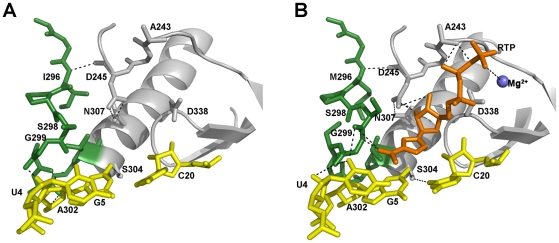
The structure and interactions of the FMDV 3D active site in two different complexes. (A) 3D(SSI)-RNA template/primer and (B) 3D(wild type)-RNA-ribavirin-triphosphate (RTP) (PDB: 2E9R). The polymerase residues in the active site are shown in grey with the loop β9-α11 highlighted in green. The first base pairs of the template/primer RNA are shown in yellow with the incoming RTP molecule in orange (in B). When the RTP molecule is located at the active site of the wild type 3D, the β9-α11 loop changes its conformation to accommodate the nucleoside analogue into the cavity, and the ribavirin pseudo-base appears hydrogen bonded to residues S298 and G299 within the loop. The side chains of residues Asp245 of motif A and Asn307 of motif B have also changed their rotamer conformations to facilitate the interactions with the ribose moiety of the mutagenic nucleotide. Substitution M296I seems to prevent the mentioned conformational changes in the loop β9-α11 as well as the side chain rearrangements in residues Asp245 and Asn307 required to interact with ribavirin.

The 3D(P44S, M296I)-RNA and 3D(SSI)-RNA structures are almost identical (rmsd of the superimposition of all polymerase residues of 0.22Å). These structures are also similar to the structure of 3D(M296I)-RNA mutant complex determined previously (PDB 3KOA; [51]), and to the wild type 3D-RNA complex (PDB 1WNE; [63]), with rmsds of 0.33Å and 0.38Å, respectively. Compared to the wild type 3D, two significant changes are observed in the substituted 3Ds: a conformational change in loop β9-α11 (where substitution M296I lies) and a structural rearrangement of the N-terminus of the polymerase. The conformation and interactions of loop β9-α11 are identical in the 3D(P44S, M296I) and 3D(SSI) complexes, retaining the same structure that was previously observed in 3D (M296I) in complex with RNA [51]. All mutants that contain the substitution M296I show a rearreagement in the loop β9-α11, consisting in a rotation of the peptide bonds Ser298-Gly299 and Cys300-Ser301 (Figure 7). These residues were found hydrogen bonded to the incoming RTP molecule in the structure of the ternary complex between the wild type 3D-RNA-RTP [65], and also interacting with the template acceptor nucleotide in all structures analyzed [51], [65], [66] (Figure 7).

**Figure 8:**
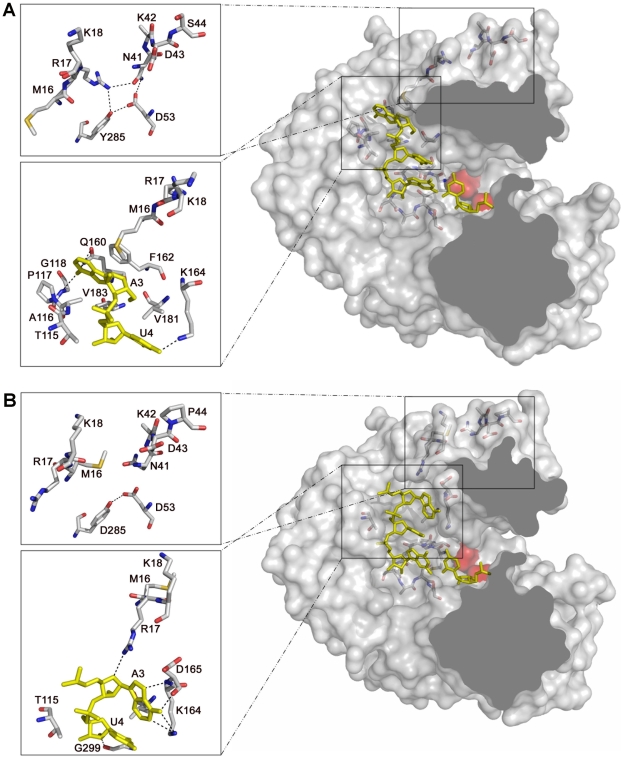
Structure and interactions in the template channel of the FMDV 3D polymerase. (A) the 3D(SSI)RNA mutant complex and (B) the 3DWt-RNA complex (PDB 1WNE). The molecular surface of the polymerase is shown in grey with the acidic residues of the active site in red and the RNA depicted as a ribbon in yellow. Only the 5′ overhang moiety and the first base pair in the active site is shown for clarity. Residues of the β2-α2 loop (containing S44), the amino acid interacting with β2-α2 loop and, those contacting the RNA template are shown as sticks in atom type colour. The left side insets in A and B show close-ups of the interactions involving the loop β2-α2 (top) and template nucleotides A3 and U4 (bottom).

**Figure 9:**
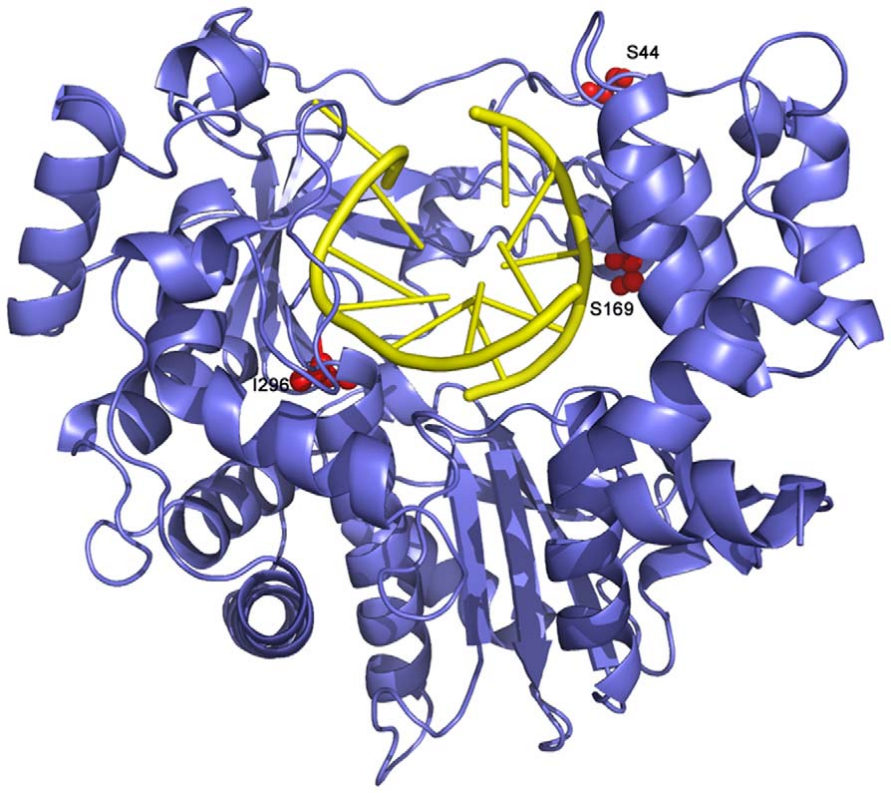
Ribbon diagram of the structure of FMDV 3D polymerase, SSI mutant, in complex with the RNA template-primer. The polymerase is depicted in blue and the RNA in yellow. The substituted amino acids, S44, S169 and I296, are shown as red balls and explicitly labelled.

Interestingly, the amino acid residues from M16 to K18, at the N-terminus of the enzyme, appear totally re-organized (Figure 8). This region, together with residues T115 to A122 of motif G and amino acids Q160, F162 and T181 of motif F, form the template channel that binds the 5′ overhang region of the template, driving the ssRNA to the active site cavity [64]. The structures of the wild type 3D-RNA elongation complexes as well as the structure of the mutant 3D(M296I)-RNA complex show that R17 interacts with the sugar-phosphate backbone of template nucleotide A3 that is oriented towards the active site cavity (Figure 9; [64], [65]). In 3D(P44S, M296I)-RNA and 3D(SSI)-RNA complexes the re-oriented residue R17 points to the polymerase interior, interacting with the side chain of residues N41 (which lies in the same loop of the substituted amino acid S44), and with Y285. Nucleotide A3 appears also reoriented, flipped-out towards a pocket formed by amino acids M16, P117, G118, Q160, F162, V181 and V183 (Figure 8). These structural results indicate that the small movements in the loop, that contains the substituted residue S44, facilitate the large conformation changes of the 3D N-terminal residues M16-K18, and the reorientation of the template nucleotide A3. The rearrangements in M16-K18 were also observed in the uncomplexed 3D(P44S), but with a weak electron density and higher temperature factors than the average, reflecting some degree of flexibility of this region in the absence of RNA.

Finally, no significant structural changes were observed associated with substitution P169S. Thus, the structural results point at P44S as the key substitution related to reorientation of template residues that might be associated with altered RMP recognition and incorporation.

## Discussion

The great adaptive capacity of RNA viruses to adverse environmental conditions has been fully manifested in the present study with the selection of mutant polymerases capable of biasing the incorporation of RMP so as to modulate the overall mutation types in progeny genomes. The adaptation of FMDV to high R concentrations was mediated by the sequential selection of M296I, P44S and P169S in 3D, with P44S being the main responsible for maintaining a balance of transition types in progeny RNA synthesized in the presence of R. The three amino acid substitutions in 3D were the result of mutation types that are favored during replication of FMDV in the presence of R: a G→A transition in the case of M296I, and a C→U transition in the case of P44S and P169S. Except for P44S and P169S when present individually in 3D, the substitutions in 3D had as consequence a modest but consistent decrease in viral fitness when measured in the absence of R. None of the three replacements in 3D has been previously observed in FMDV C-S8c1 populations (or their mutant spectra) passaged in the absence of R or in the presence of FU or 5-azacytidine [16], [27], [42], [53], [58]–[60], [67]–[71]. Thus, they were selected as a specific response to R and, as expected, the combination of the three substitutions increased FMDV fitness during virus replication in the presence of R (Table 4). Remarkably, the selective advantage of FMDV expressing 3D with the triple combination P44S, P169S, M296I over virus expressing 3D with P44S and M296I was manifested in growth-competition experiments carried out in the presence of 5000 µM R but not in the presence of 800 µM R. Thus, P169S appears to have been selected to contribute a fitness increase in the presence of high R concentrations to a virus that had already acquired the capacity to modulate the mutational spectrum through substitution P44S in 3D. Additional growth-competition experiments between wild type and the triple mutant FMDV indicated that the substituted polymerase conferred a selective advantage when the virus replicated in the presence of R but not of FU or a mixture of R and FU, supporting a specific adaptative response in front of ribavirin (Table 6). The result is consistent with the fitness advantage of R-Ap60 over Ap35 in the presence of R but not of FU+GuH (Table 2). Since FU tends to evoke the opposite transition types than R [58], [60], the outcome of the competitions reinforces modulation of transition types as a major factor for the survival of FMDV 3D(SSI) in the presence of ribavirin.

It may be argued that selection of the multi-substituted polymerase occurred as a result of subjecting the virus to extremely large ribavirin concentrations, unlikely to be reached during any standard antiviral treatment with R. Certainly, the concentrations used were not intended to reproduce actual R concentrations in the course of treatments with R in clinical practice. In the case of direct aerosol application of R to the upper respiratory tract the drug may reach intracellular concentrations of around 800 µM [72], [73]. Other modes of administration are unlikely to achieve such high concentrations. For example, intravenous administration of R results in peak concentrations in the range of 20 µM to 150 µM [74], [75] while oral administration resulted in concentrations between 10 µM and 20 µM in serum and cerebrospinal fluid [74], [76]–[78]. Thus, unless procedures for targetted delivery of R to specific cells or tissues are developed, it is unlikely that concentrations equivalent to those used in our experiments would be encountered *in vivo*. Do the high concentrations of R used in our experiments weaken the relevance of the conclusions? We think not for two reasons: (i) the actual concentration of RTP in the replication complexes of viruses is unknown, and it cannot be excluded that methods of targetted delivery could be developed that result in high local RTP concentrations; (ii) extreme environmental conditions (a prolonged plaque-to-plaque passage regime, passages in the presence of monoclonal antibodies, etc.) have previously been used to unveil either evolutionary responses or the sensitivity of biochemical processes to subtle genetic change [1], [69], [79]. Thus, our model studies must be regarded as designs to disclose potential mutagen-resistance mechanisms that are informative of the potential of the polymerase to adapt its catalytic machinery to extraneous substrates, despite using conditions unlikely to be encountered *in vivo*.

The virological and biochemical evidence presented here support the hypothesis that the polymerase substitutions, whose effect was to avoid a highly biased distribution of mutation types normally induced by a mutagenic agent, contributed to viral survival and escape from extinction (Figure 3), implying a new mechanism of virus resistance to lethal mutagenesis. This new mechanism does not require significant reductions of mutant spectrum complexity thereby preserving an amplitude of the mutant cloud adequate for virus adaptability to complex environments or following a bottleneck event [1], [28]–[30]. The balanced mutational spectrum produced by FMDV 3D(SSI) was maintained in the absence or presence of 5000 µM R, while FMDV Wt produced a mutant spectrum with 80% (C→U)+(G→A) only after 4 passages in the presence of 5000 µM R (Table 5). A deleterious effect of biased substitution types is likely because they can affect codon usage and specific RNA structures needed during viral replication [80]–[85]. Examination of the repertoire of mutations (and corresponding amino acid substitutions) present in the mutant spectra of FMDV Wt and FMDV SSI passaged in the presence of 5000 µM R is highly illustrative of the deleterious effects of the incapacity of the polymerase to modulate transition types (Table 9). First, the proportion of non-synonymous mutations relative to the number of nucleotides sequenced is 1.5-fold higher for R-Wt-4 than for R-SSI-4. Second, in the R-Wt-4 population a stop codon was generated as a result of a G→A transition at genomic position 7319, and 74% of the 27 amino acid substitutions scored were the result of C→U or G→A transitions. In contrast, of the 15 amino acid substitutions in R-SSI-4 only 20% were the result of C→U or G→A transitions. The most salient amino acid substitutions found in population R-Wt-4 are G125R, C300Y and G435E, each originated from a G→A transition (Table 9). These residues are conserved among picornaviruses and the substitutions observed might have relevant structural effects. G125R is an infrequent substitution that introduces a bulky residue that was tolerated probably because it lies in an exposed region at the entrance of the template channel [63], [64]. C300 is located in loop β9-α11, and its main chain interacts with the rNTP and the acceptor base of the template RNA. In the complex with RTP, the G299-C300 peptide bond is rotated in a way that favors the interaction with the pseudobase [65] (Figure 7). Replacement of C by Y is likely to affect the flexibility of this region and, as a result, the interactions with RNA and the rNTP. G435 is located in a short turn between helices α14 and α15 in the thumb domain, a region which is rich in small and flexible amino acids [63]. The introduction of an E residue in this region is not expected, and it might affect the stability of this 3D region. In contrast to R-Wt-4, among the amino acid replacements found in the R-SSI-4 populations, the most noticeable is K164E located in motif F of 3D. K164 is not among the basic amino acids that interact with the incoming rNTP, but it is hydrogen bonded to template base A3 [63]. An E residue could participate in the same interaction, as also observed between 3D residue D165 and base U4 in the same complex [63]. Thus, the comparison of the mutant repertoire in R-Wt-4 and R-SSI-4 reinforces the likely adverse effects of an abundance of C→U and G→A transitions for FMDV fitness. It is not clear whether the most detrimental factor is the imbalance of mutation types by itself, or the increased frequency of U and A residues in genomic RNA, or a combination of both factors. Whatever the mechanism, the results suggest that the maintenance of a suitable transition pattern during RNA synthesis in an environment of high mutational pressure can be beneficial for the virus under increased average mutation rates.

**Table 9:** Mutations and corresponding amino acid substitutions in the mutant spectra FMDV R-Wt-4 and R-SSI-4 passaged in the presence of ribavirin^a^.

R-Wt-4		R-SSI-4	
Mutation^b^	Amino acid^b^	Mutation^b^	Amino acid^b^
C6680T	-	C6765T	**-**
G6696A	**-**	T6683C	**V25A**
C6719T	**A37V**	G6836A	**R76H**
C6720T	**-**	A6858G	**-**
C6722T	**A38V**	T6888C	**-**
T6727C	**S40P**	T6888C	**-**
G6760A	**V51I**	A6912G	**-**
C6763T	**L52F**	T7010C	**F134S**
T6773C	**V55A**	A7076C	**K156T**
T6923C	**V105A**	A7099G	**K164E**
C6975T	**-**	A7100G	**K164R**
G6982A	**G125R**	T7110C	**-**
G6989A	**R127H**	C7134T	**-**
T7083C	**-**	G7140A	**-**
G7120A	**E171K**	G7165A	**V186I**
T7193C	**M195T**	T7206C	**-**
G7202A	**R198K**	A7212G	**-**
C7230T	**-**	T7241C	**I211T**
G7236A	**-**	T7344C	**-**
G7319A	**STOP**	T7367C	**M253T**
C7353T	**-**	G7416A	**-**
A7375G	**M256V**	A7417G	**N270D**
G7420A	**A271T**	C7432T	**-**
C7431T	**-**	C7479T	**-**
C7443T	**-**	C7506T	**-**
A7448G	**N280S**	A7515G	**-**
G7473A	**-**	A7522G	**I305V**
G7508A	**C300Y**	C7665T	**-**
C7548T	**-**	C7697T	**T363I**
C7562T	**A318V**	T7757C	**V383A**
G7633A	**V341M**	T7868C	**I420T**
G7654A	**D349N**	T7904C	**V432A**
G7663A	**A352T**	A7917G	**-**
C7664T	**A352V**	C7986T	**-**
C7755T	**-**	A7980G	**-**
T7841C	**I411T**		
G7856A	**R416H**		
G7862A	**G418E**		
G7863A	**-**		
G7872A	**-**		
G7891A	**A428T**		
G7913A	**G435E**		
C7926T	**-**		
C7933T	**L442P**		
**Total mutations** c	**44**	**Total mutations**	**35**
**G→A+C→T** d	**21**	**G→A+C→T**	**3**
**Synonymous (%)** e	**16 (36)**	**Synonymous (%)**	**20 (57)**
**Non-synonymous (%)** e	**28 (64)**	**Non-synonymous (%)**	**15 (43)**

a The sequence of the 3D-coding region was determined for R-Wt-4 and R-SSI-4 (wild type and triple mutant FMDV) after 4 passages in the presence of 5000 µM ribavirin. The populations are those described in Figure 3 and Table 5.

b Mutation and deduced amino acid substitutions are relative to the sequence of the parental clone C-S8c1 [57]. Amino acid residues (single-letter code) are numbered from the N- to the C-terminus of 3D. Boldface type indicates a change in the amino acid residue. Procedures for nucleotide sequencing and identification of FMDV genomic regions are described in Materials and Methods.

c Number of different mutations found comparing the sequence of each individual clone with the corresponding sequence of FMDV C-S8c1 [57].

d Number of G→A plus C→T substitutions found comparing the sequence of each individual clone with the corresponding sequence of FMDV C-S8c1 [57].

e The percentage of synonymous and non-synonymous mutations is indicated in parenthesis.

Despite the clear virological and biochemical effects of substitutions P44S, P169S and M296I in 3D, the comparison of fitness values for clones and populations indicates that it is unlikely that the replacements in 3D are the only determinants of high level resistance to R. Indeed, the fitness of the uncloned FMDV population R-Ap60 was 15-fold higher than the fitness of control population Ap35, when measured in the presence of 5000 µM R, while the fitness of the cloned FMDV 3D(SSI) was 2-fold higher than the fitness of the cloned FMDV Wt, measured under the same conditions (Tables 2 and 4). There are two main possibilities to account for the larger difference of fitness between FMDV R-Ap60 and Ap35 than between FMDV 3D(SSI) and FMDV Wt. One is that the complexity or composition of the mutant spectrum of R-Ap60 conferred a selective advantage to the mutant ensemble that could enhance R resistance, even in the absence of additional dominant mutations (or mutations in their way to dominance). Recent observations on the selective value of mutant spectrum complexity and composition [1], [29], [53], [71], [86], [87] do not permit excluding this possibility. An alternative, not mutually exclusive possibility, is that mutations in genomic regions of FMDV other than 3D contribute also to R resistance in RAp60. Current evidence suggests that non-structural protein 2C may also contribute to FMDV escaping extinction (Agudo et al., manuscript in preparation).

P44 is conserved among known picornaviral polymerases, and it lies in a loop that connects strand β2 and helix α2 in the fingers domain (Figure 9). This loop contains a number of residues that establish tight contacts with amino acids V173 to G176 of motif F and with the N-terminal residues M16, R17 and K18 of 3D. Amino acids M16 and R17 form part of the template channel that drives the ssRNA template towards the active site. Thus, substitutions at the conserved amino acid P44 might disturb both the shape and interactions of the template channel, and the interactions with the incoming rNTP that are mediated by residues of motif F.

The structures of the mutant polymerases determined in the present study do not show large domain movements. However, the crystal structures of 3D(P44S, M296I) and 3D(SSI) in complex with the RNA template-primer reveal a rearrangement in the template channel with important effects in template binding, in particular, at position n+1 (nucleotide A3). The conformational changes in the main and side chains of residues M16 and R17 allow the opening of a hydrophobic pocket formed by residues of motifs G and F and by M16 that facilitates the entrance of nucleotide A3 (Figure 8). The polymerase with substitution M296I that acquired substitution P44S maintained the alteration of loop β9-α11 previously described for 3D (M296I) [51] (Figure 8). Thus, the catalytic domain and template interactions may be affected by additive effects of substitutions M296I and P44S. The different interactions established between the modified template channel of the substituted polymerases and nucleotide A3 could facilitate a different alignment of the template strand, thus altering the nucleotide incorporation activity. However, this possibility has not been substantiated because of the inability of nucleotide substrates to be incorporated into the mutant 3D-RNA complexes.

Finally, P169 is a non-conserved residue located in motif F of 3D (Figure 9) that has been implicated in the recognition of the triphosphate moiety of the incoming nucleotide. P169 is close to 3D residues that directly contact with either the triphosphate or ribose moieties of the incoming nucleotide [63], [88]. The structural comparisons do not reveal any conspicuous change in the polymerase induced by substitution P169S. However, we can not exclude that a change at this position could also affect the recognition of an incoming nucleotide, modulating its incorporation rate, and thus altering the replication fidelity or replicative fitness. Thus, subtle structural modifications that affect the template channel of 3D mediate alterations in substrate recognition that may modify recognition of RTP and the repertoire of R-mediated mutations.

## Materials and Methods

### Cells, viruses, infections, and cytotoxicity of ribavirin

The origin of BHK-21 cells, procedures for cell growth and for infection with FMDV in the presence or absence of ribavirin (R; Sigma), 5-fluorouracil (FU; Sigma), or guanidine hydrochloride (GuH; Sigma) have been previously described [27], [53], [54], [59]. Briefly, for each infection the first passage was carried out at moi 0.3 PFU/cell. For the following passages, 2×10^6^ BHK-21 cells were passaged with supernatant of virus from the previous passage (0.2 ml), and the infection allowed to proceed for about 24h. Values of PFU for each passage can be estimated from infectivities given in Figure 3. FMDV C-S8c1 is a plaque-purified derivative of natural isolate C1 Santa-Pau Spain 70 [54], a representative of European serotype C FMDV. FMDV MARLS is a monoclonal antibody-escape mutant selected from the C-S8c1 population passaged 213 times in BHK-21 cells [69]. Ap35 and R-Ap35 are FMDV MARLS passaged 35 times in the absence or in the presence, respectively, of increasing concentrations of R as previously described [27]; FMDV MARLS populations passaged 45 and 60 times in the presence of increasing concentrations of R have been termed R-Ap45 and R-Ap60 (see Figure 1). R exerted a cytostatic effect in BHK-21 cell monolayers (measured as cell viability using trypan blue staining). The cytotoxicities as a result of treatment of BHK-21 cell monolayers with R, FU and GuH have been previously described [53], [58], [59]. The maximum reduction of cell viability of confluent BHK-21 cell monolayers in the presence of 5000 µM R was around 40% at 48 h post-treatment, in agreement with our previous results [53]. Evidence that cytotoxicity by R does not contribute significantly to FMDV extinction includes the observation that FMDV mutant with amino acid replacements in 3D that confer resistance to R can replicate and survive after multiple passages in the presence of 5000 µM R (Figure 3, and unpublished observations).

### Extraction of RNA, cDNA synthesis, PCR amplification, and nucleotide sequencing

RNA was extracted from the supernatants of infected cells using described procedures [27], [67]. Reverse transcription (RT) was carried out using AMV reverse transcriptase (Promega), and PCR amplification was performed using EHF DNA polymerase (Roche) as specified by the manufacturer. RT-PCR amplification intended for the cloning of individual cDNA molecules was carried out using *Pfu ultra* DNA polymerase (Stratagene). Amplification protocols, nucleotide sequencing and primers used for amplification and sequencing have been previously described [27], [42], [67].

### Quantification of viral RNA

FMDV RNA was quantified by real-time RT-PCR amplification using the Light Cycler instrument (Roche) and the RNA Master SYBR green I kit (Roche) as previously described [27].

### Preparation of FMDV C-S8c1 with substitutions in 3D

Plasmid pMT28 encodes an infectious transcript of FMDV C-S8c1 [57], [70]. The construction of plasmid pMT28-3D(M296I) (an infectious clone expressing 3D with substitution M296I in the context of the C-S8c1 genome) has been previously reported [27]. The rest of chimeric plasmids encoding mutant 3Ds were constructed by replacing part of the 3D-coding region of pMT28 with the corresponding mutant 3D-coding region of interest. To construct pMT28-3D(P169S) (an infectious clone encoding 3D with amino acid substitution P169S in the context of the C-S8c1 genome), two DNA amplifications were carried out using *Pfu ultra* DNA polymerase and pMT28 DNA as template. A first amplification with 3AR3 (GATGACGTGAACTCTGAGCCCGC; sense, 5′ position 5710) and 3′3DP169S (CTTTCTCCAT**GCT**GCGAATTTCGTCCTTCAGGAAGG; antisense, 5′ position 7126); and a second amplification with 5′3DP169S (CGAAATTCGC**AGC**ATGGAGAAAGTACGTGCCGG; sense, 5′ position 7104) and 3D1 (CTTGTTGCGGAACAGCCAGATG; antisense, 5′ position 7520) were performed (bold-face letters indicate modifications of the genomic sequence introduced to express 3D with substitution P169S). (Nucleotide positions correspond to the numbering of FMDV genomic residues described in [69]). The two amplicons were shuffled and digested with *Rsr*II (position 5839) and *Cla*I (position 7004) (New England Biolabs) and ligated to pMT28 DNA linearized with the same enzymes. A similar procedure was used to construct pMT28-3D(P44S) and pMT28-3D(P44S, M296I). To prepare pMT28-3D(P44S,M296I) (an infectious clone expressing 3D harboring substitution P44S and M296I in the context of the C-S8c1 genome), pMT28-3D(M296I) was subjected to the same procedure described above for pMT28, except that the two pairs of primers used for the PCR amplification were 3AR3 (described above) with 3′3DP44S (CGTTCAGACG**GCT**GTCCTTGTTAGACAAGGCGG; antisense, 5′ position 6751), and 5′3DP44S (CTAACAAGGAC**AGC**CGTCTGAACGAAGGTG; sense, 5′ position 6728) with A3 (CGTCGACAATGCGAGTCTTGCCG; antisense, 5′ position 7156; bold-face letters indicate modifications of the genomic sequence introduced to express 3D with substitution P44S). The two amplicons were shuffled, digested with *Rsr*II and *Cla*I, and ligated to pMT28 or pMT28-3D(M296I) DNAs linearized with the same enzymes, rendering pMT28-3D(P44S) and pMT28-3D(P44S, M296I), respectively. Finally, to construct pMT28-3D(P44S, P169S, M296I) (an infectious clone expressing 3D with amino acid substitution P44S, P169S and M296I in the context of the C-S8c1 genome), procedures were carried out as those described for pMT28-3D(P169S) except that the parental plasmid used both as template for DNA amplifications and for cloning was pMT28-3D(P44S, M296I) instead of pMT28. For simplicity, the plasmid that includes the three amino acid substitutions in 3D has been termed pMT28-3D(SSI) and the rescued virus FMDV 3D(SSI).

Ligation, transformation of E. *coli* DH5α, colony screening, nucleotide sequencing, preparation of infectious RNA transcripts, and RNA transfections were carried out as previously described [27], [67].

### Quasispecies analysis

To determine the complexity of mutant spectra, FMDV RNA was extracted as described above and subjected to RT-PCR using primers PolC-KpnI (GTT*GG* ***T****AC****C***CACTCTGCTGGAGGC; sense, 5′ position 6502) and Pol1-XbaI (AA*T* ***CTA****GA*TGTTTGGGGGATTATGCG; antisense, 5′ position 8060; the letters underlined indicate the sequences recognized by restriction enzymes *Kpn*I and *Xba*I, respectively). cDNA was digested by *Kpn*I and *Xba*I enzymes (New England Biolabs), and ligated to plasmid pGEM-3Z Vector (Promega) previously digested with the same enzymes. Transformation, colony screening and nucleotide sequencing were carried out as previously described [27], [67]. The region sequenced spans residues 6508 to 8036 and includes the entire 3D-coding region (residues 6610 to 8020). The number of clones analyzed and the total number of nucleotides sequenced are given in the appropriate section of Results. The complexity of mutant spectra was expressed as the mutation frequency, calculated by dividing the number of different mutations by the total number of nucleotides sequenced.

### Fitness assays

Relative fitness was measured by growth-competition experiments in the presence or absence of R. The logarithm of the ratio of the two competing viruses was plotted against passage number, and the fitness vector was adjusted to an exponential equation ***y*** = a×e^b***x***^. The antilogarithm of the vector slope is the fitness of the virus tested, relative to that of the reference virus [27], [68]. The proportion of the two competing genomes at different passages was determined by real-time RT-PCR, employing primers specifically designed to discriminate accurately the two RNAs in the competition (Table 10). For each fitness determination, the R^2^ value of the corresponding linear regressions is also given (Tables 2 and 4).

**Table 10:** Primers used to determine the relative fitness of FMDV populations.

Primer	Population^a^	Sequence^b^	Sense^c^	C°^d^	Position^e^	Primer pair^f^
**Mk Wt**	pMT28Ap35	GGAACAGCCAGATGG**C**AT	R	72	7512	3DR4
**Mk Res**	pMT28-3D(M296I)R-Ap35R-Ap60	GGAACAGCCAGATGG**T**AT	R	68	7512	3DR4
**P169**	pMT28pMT28-3D(M296I)pMT28-3D(2M)	CCTGAAGGACGAAATTCGC**CCG**	F	63	7095	AV3
**S169**	pMT28-3D(3M)	CCTGAAGGACGAAATTCGC**AGC**	F	63	7095	AV3
**3DR4**	All populations	ACTCGCATTGTCGACGTTTT	F	--	7141	--
**AV3**	All populations	TTCATGGCATCGCTGCAGTGG	R	--	7370	--

a Viral populations which are specifically discriminated by the primer indicated in b.

b Nucleotide sequence of the primers used. Letters in bold are nucleotides that have been modified with respect to the genomic sequence of the FMDV C-S8c1, and that serve to discriminate among different populations.

c Genomic orientation of primer: forward (F) or reverse (R).

d Hybridization temperature used in the real time RT-PCR assays to discriminate the competing RNAs. “--” indicates that this primer does not discriminate specifically among mutant genomes.

e Position of the 5′-nucleotide of the primer; numbering is that described in [93].

f Complementary primer used in each case during RT-PCR amplification. “--” indicates that this primer does not discriminate specifically among any mutant genomes and is used as the complementary for the RT-PCR reaction.

### Fitness assays in the presence of ribavirin, 5-fluorouracil and a mixture of both drugs

A solution of ribavirin (R) in PBS was prepared at a concentration of 100 mM, sterilized by filtration, and stored at −70°C. Prior to use, the stock solution was diluted in DMEM to reach the desired R concentration. To prepare culture medium containing 5-fluorouracil (FU) (Sigma), the analogue was dissolved in DMEM to yield a 5 mg/ml solution, and diluted in DMEM, as needed for the experiments. For infections in the presence of R (5000 µM) and FU (2000 µM), cell monolayers were treated during 7 h and 10 h, respectively, prior to infection. The relative fitness of FMDV SSI was determined by growth competition with the Wt virus in BHK-21 cells in the presence of R, FU or a mixture of both drugs. Briefly, the viral population to be assayed was mixed with the same number of PFU of FMDV Wt, used as reference. For each determination, four serial infections were carried out at moi 0.3 PFU/cell. The proportion of the two competing genomes at each passage was determined by measuring the area of the three peaks corresponding to the residues that distinguish 3D Wt from 3D(SSI). Each mutation was confirmed by two independent sequencing assays using primers of different orientation. The average of triplicate measurements and standard deviations are given.

### Molecular cloning, expression, and purification of FMDV 3D

FMDV 3D with substitutions M296I [termed 3D(M296I)], P169S [termed 3D(P169S)], P44S [termed 3D(P44S)], with P44S and M296I [termed 3D(P44S, M296I)], or with the three of them [3D(P44S, P169S, M296I) which is abbreviated as 3D(SSI)] were obtained from plasmid pET-28a 3Dpol [expression vector pET-28a (Novagen) containing the FMDV polymerase 3D-coding region [67]] by site-directed mutagenesis with oligonucleotides containing the corresponding mutated nucleotides, using the QuickChange site-directed mutagenesis kit (Stratagene). Mutagenesis, 3D expression, and 3D purification by affinity chromatography, were carried out as previously described [63], [67]. The enzymes were >95% pure, as judged by SDS-PAGE electrophoresis and Coomassie brilliant blue staining.

### 3D-polymerization assays using heteropolymeric template-primers

Incorporation of standard nucleoside-5′-triphosphates or ribavirin-5′-triphosphate (RTP) by wild type and mutant 3Ds was measured in self-complementary RNAs that form double stranded RNA in which each strand can act both as template and primer [62]. RNAs 5′-CGUAGGGCCC-3′ (termed sym/sub-AU), 5′-UGCAGGGCCC-3′ (termed sym/sub-AC), 5′-GUACGGGCCC-3′ (termed sym/sub-C) and 5′-GCAUGGGCCC-3′ (termed sym/sub-U) (Dharmacon Research) were used. The oligonucleotides were purified, end-labeled with [γ-^32^P] ATP and polynucleotide kinase (New England biolabs), and annealed using standard protocols [27], [89]. For the reaction with sym/sub-AU and sym/sub-U, 0.5 µM of RNA-duplex and 2 µM 3D were incubated in 30 mM MOPS (pH 7.0), 33 mM NaCl, 5 mM Mg(CH_3_COO)_2_, and 50 µM UTP (Amersham) in the case of sym/sub-AU, for either 10 min [for 3D *wt*, 3D(P169S) and 3D(M296I)] or 30 min [for 3D(P44S) and 3D(SSI)] at 37°C, or 33°C when mentioned; 3D(SSI) and 3D(P44S) were incubated for longer periods of time because they display a defect in RNA binding (see Results). After formation of a binary complex of 3D-RNA, [elongated in one nucleotide in the case of sym/sub-AU (3D-sym/sub-AU, n+1 complexes)], an excess of unlabeled sym/sub-AU (5 µM) was added to trap the unbound 3D, and to avoid the recycling of labelled sym/sub-AU. The reaction was initiated by adding either 50 µM ATP (Amersham) or 50 µM (RTP) (Moraveck), or 1µM ATP when mentioned. The reaction was stopped at different times by the addition of EDTA (83 mM final concentration). Identical procedure was followed with sym/sub-AC, except that GTP and RTP were used as substrates. Reaction products were analyzed by electrophoresis on a denaturing 23% polyacrylamide, 7 M urea gel in 90 mM Tris-base, 90 mM boric acid, 2 mM EDTA. The 11 mer (sym/sub elongated in one nucleotide by addition of UMP) and ≥12-mer (sym/sub elongated in two or more nucleotides by addition of the required nucleotides) were visualized and quantitated with a Phosphorimager (BAS-1500; Fuji).

### Other assays with 3D

Poly(rU) synthesis using poly(A)-oligo(dT)_15_ as template-primer molecule, VPg uridylylation with poly(A) as template and Mn^++^ as ion, and RNA binding assays were carried out as previously described [67], [89].

### Co-crystallization experiments

Purified FMDV mutant polymerases 3D(P44S), 3D(P169S), 3D(P44, M296I) and 3D(SSI) were stored in a buffer containing Tris-HCl (40mM, pH 7.5), NaCl (0.5M), DTT (0.8mM), EDTA (0.8mM), and glycerol (8%), at a concentration of ∼4.6 mg/ml. The oligonucleotide 5′GCAUGGGCCC 3′ (NWG-Biotech) (sym/sub-U) was annealed following the described procedure [62]. Then the 3D was added slowly to an equimolar proportion in the presence of 2mM MgCl_2_. The mutant 3Ds and their complexes were crystallized as previously described [63].

### Data collection, structure determination and refinement

Four different data sets were collected at 100 K: 3D(P44S) (2.2 Å), 3D(P169S) (2.6 Å), 3D(P44, M296I)-RNA (2.6 Å) and 3D(SSI)-RNA (2.5 Å), using synchrotron radiation at the ESRF beamlines ID14 EH1 and EH2 (λ = 0.93 Å). All data were processed and reduced using DENZO/SCALEPACK package [90] (Table 8).

The initial maps for the 3D(P44S) and 3D(P169S) (tetragonal crystals) were obtained after a rigid body fitting of the coordinates of isolated 3D protein that was crystallized in the tetragonal p4_2_2_1_2 space group (PDB:1U09) [63] to the new unit cells, using the program REFMAC (CCP4). Initial maps for the 3D(P44, M296I)-RNA and 3D(SSI)-RNA complexes (P3_2_21 crystals) were obtained following the same procedure but using the trigonal P3_2_21 coordinates of 3D (PDB:1WNE) [63] as starting model (Table 1). In the four structures the 2|Fo|-|Fc| and |Fo|-|Fc| difference maps clearly allowed the re-positioning of the mutated residues and surrounding regions and, in the trigonal structures, these maps showed the presence of extra densities corresponding to the RNA template-primers. However, the tetragonal crystals, 3D(P44S) and 3D(P169S), did not contain RNA despite using the same incubation and co-crystallization conditions as in 3D(P44, M296I)-RNA and 3D(SSI)-RNA complexes that crystallized in the P3_2_21 space group. Several cycles of automatic refinement, performed with program REFMAC, were alternated with manual model rebuilding using the graphic programs TURBO and Coot [91]. The statistics of the refinement for the four complexes are summarized in Table 7.

## Supporting Information

Figure S1 Incorporation of nucleotides into sym/sub-AC by mutant FMDV polymerases. (A) Kinetics of incorporation of GMP into sym/sub-AC (sequence shown at the top) by the indicated FMDV polymerases [3DWt and 3D(SSI)], performed at 37°C. The reactions were initiated by addition of 1 µM GTP after the formation of 3D-RNA(n+1) complex, as described in Materials and Methods of the main text. At different time points the reaction was quenched by addition of EDTA. (B) Same as (A), except that the reaction was performed at 33°C. (C) Percentage of primer elongated to position +2, (12 mer or larger RNAs synthesized), calculated from the densitometric analysis of the electrophoreses shown in A. The results are the average of three independent experiments, and standard deviations are given. (D) Same as (C) for the incorporation of GMP at position +2 (12 mer) calculated from the densitometric analysis of the electrophoreses shown in (B). Procedures are detailed in Materials and Methods of the main text.
(7.49 MB TIF)

Figure S2 Incorporation of nucleotides into sym/sub-AU by mutant FMDV polymerases. (A) Kinetics of incorporation of AMP into sym/sub-AU (sequence shown at the top) by the indicated FMDV polymerases [3DWt and 3D(SSI)], performed at 37°C. The reactions were initiated by addition of 1 µM ATP after the formation of 3D-RNA(n+1) complex, as described in Materials and Methods of the main text. At different time points the reaction was quenched by addition of EDTA. (B) Same as (A), except that the reaction was performed at 33°C. (C) Percentage of primer elongated to position +2, (12 mer or larger RNAs synthesized), calculated from the densitometric analysis of the electrophoreses shown in A. The results are the average of three independent experiments, and standard deviations are given. (D) Same as (C) for the incorporation of AMP at position +2 (12 mer) calculated from the densitometric analysis of the electrophoreses shown in (B). Procedures are detailed in Materials and Methods of the main text.
(7.46 MB TIF)

Figure S3 Incorporation of nucleotides into sym/sub-C and sym/sub-U by mutant FMDV polymerases. (A) Kinetics of incorporation of GMP into sym/sub-C (sequence shown at the top) by the indicated FMDV polymerases [3DWt and 3D(SSI)], performed at 37°C. The reactions were initiated by addition of 1 µM GTP after the formation of 3D-RNA complex, as described in Materials and Methods of the main text. At different time points the reaction was quenched by addition of EDTA. (B) Kinetics of incorporation of AMP into sym/sub-U (sequence shown at the top) by the indicated FMDV polymerases, performed at 37°C. The reactions were initiated by addition of 1 µM ATP after the formation of 3D-RNA complex, as described in Materials and Methods of the main text. At different time points the reaction was quenched by addition of EDTA. (C) Percentage of primer elongated to position +1 (11 mer or larger RNAs synthesized), calculated from the densitometric analysis of the electrophoreses shown in A. The results are the average of three independent experiments, and standard deviations are given. (D) Same as (C) for the incorporation of AMP at position +1 (11 mer) calculated from the densitometric analysis of the electrophoreses shown in (B). Procedures are detailed in Materials and Methods of the main text.
(6.37 MB TIF)
