# Supplementary figures and images for: A Multi-Step Process of Viral Adaptation to a Mutagenic Nucleoside Analogue by Modulation of Transition Types Leads to Extinction-Escape
Source: PLoS Pathog. 2010 Aug 26;6(8):e1001072. doi: 10.1371/journal.ppat.1001072 (PMC2928812; doi:10.1371/journal.ppat.1001072)

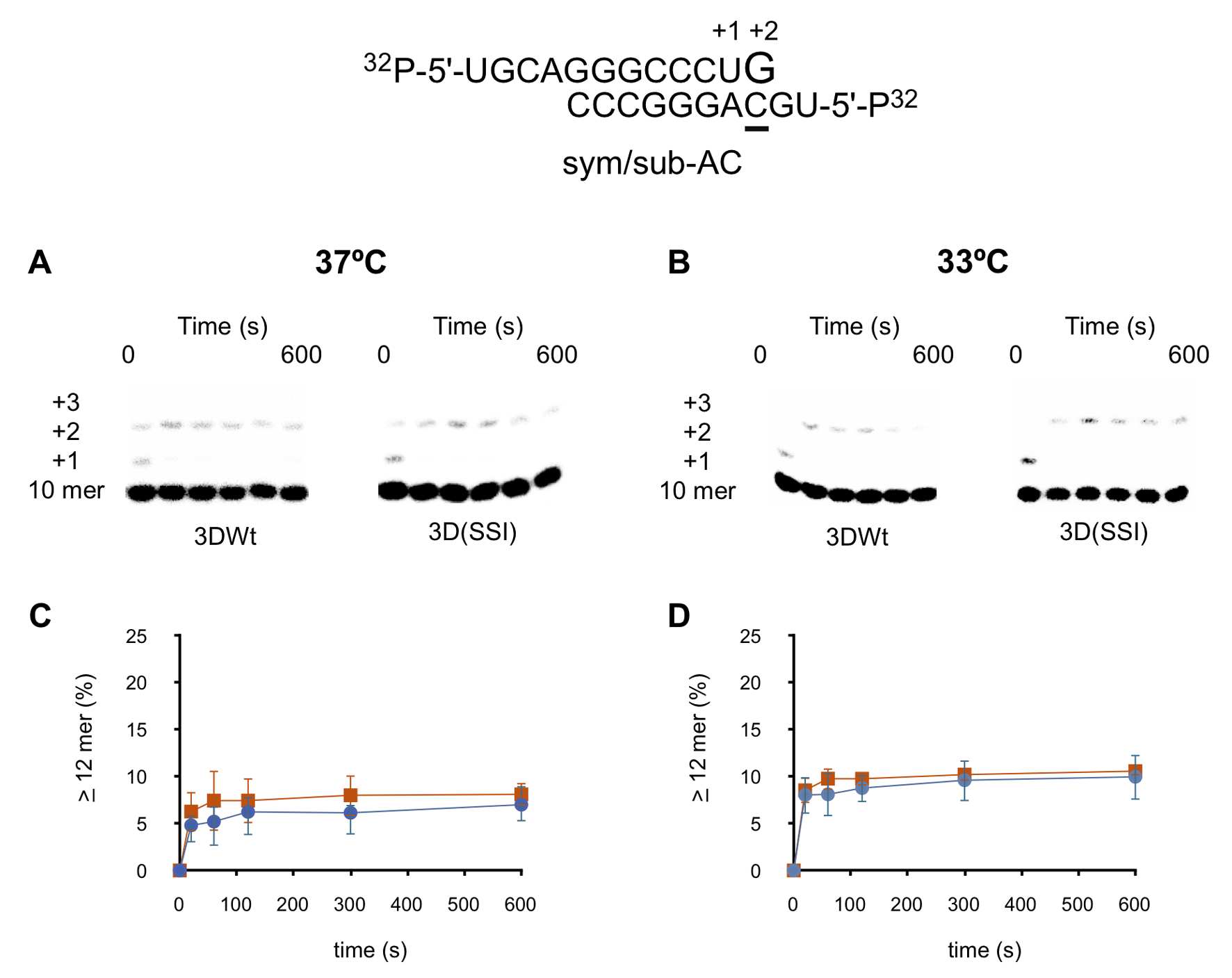

Supplement: Figure S1 — Incorporation of nucleotides into sym/sub-AC by mutant FMDV polymerases. (A) Kinetics of incorporation of GMP into sym/sub-AC (sequence shown at the top) by the indicated FMDV polymerases [3DWt and 3D(SSI)], performed at 37°C. The reactions were initiated by addition of 1 µM GTP after the formation of 3D-RNA(n+1) complex, as described in Materials and Methods of the main text. At different time points the reaction was quenched by addition of EDTA. (B) Same as (A), except that the reaction was performed at 33°C. (C) Percentage of primer elongated to position +2, (12 mer or larger RNAs synthesized), calculated from the densitometric analysis of the electrophoreses shown in A. The results are the average of three independent experiments, and standard deviations are given. (D) Same as (C) for the incorporation of GMP at position +2 (12 mer) calculated from the densitometric analysis of the electrophoreses shown in (B). Procedures are detailed in Materials and Methods of the main text. (7.49 MB TIF) [file ppat.1001072.s001.tif]

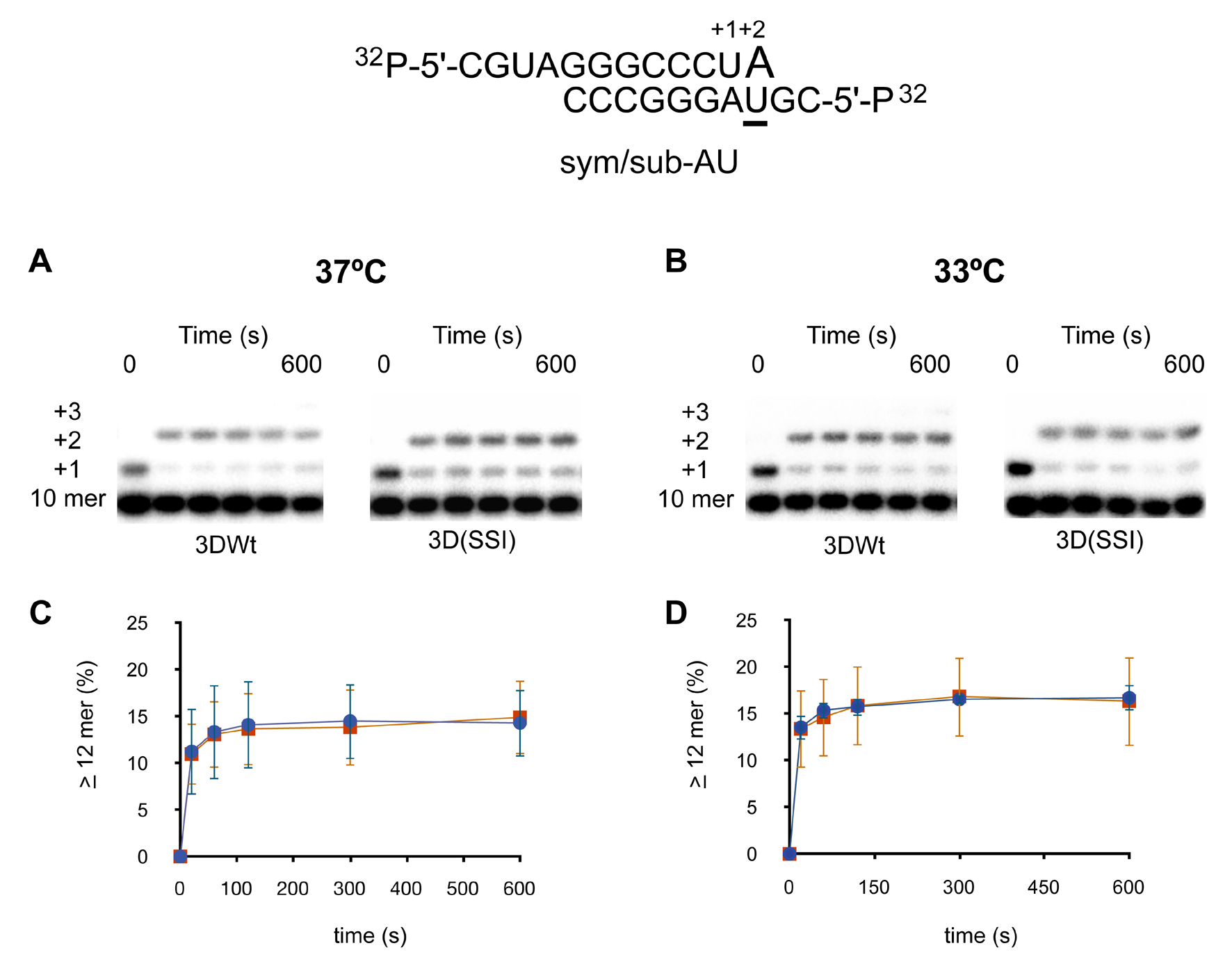

Supplement: Figure S2 — Incorporation of nucleotides into sym/sub-AU by mutant FMDV polymerases. (A) Kinetics of incorporation of AMP into sym/sub-AU (sequence shown at the top) by the indicated FMDV polymerases [3DWt and 3D(SSI)], performed at 37°C. The reactions were initiated by addition of 1 µM ATP after the formation of 3D-RNA(n+1) complex, as described in Materials and Methods of the main text. At different time points the reaction was quenched by addition of EDTA. (B) Same as (A), except that the reaction was performed at 33°C. (C) Percentage of primer elongated to position +2, (12 mer or larger RNAs synthesized), calculated from the densitometric analysis of the electrophoreses shown in A. The results are the average of three independent experiments, and standard deviations are given. (D) Same as (C) for the incorporation of AMP at position +2 (12 mer) calculated from the densitometric analysis of the electrophoreses shown in (B). Procedures are detailed in Materials and Methods of the main text. (7.46 MB TIF) [file ppat.1001072.s002.tif]

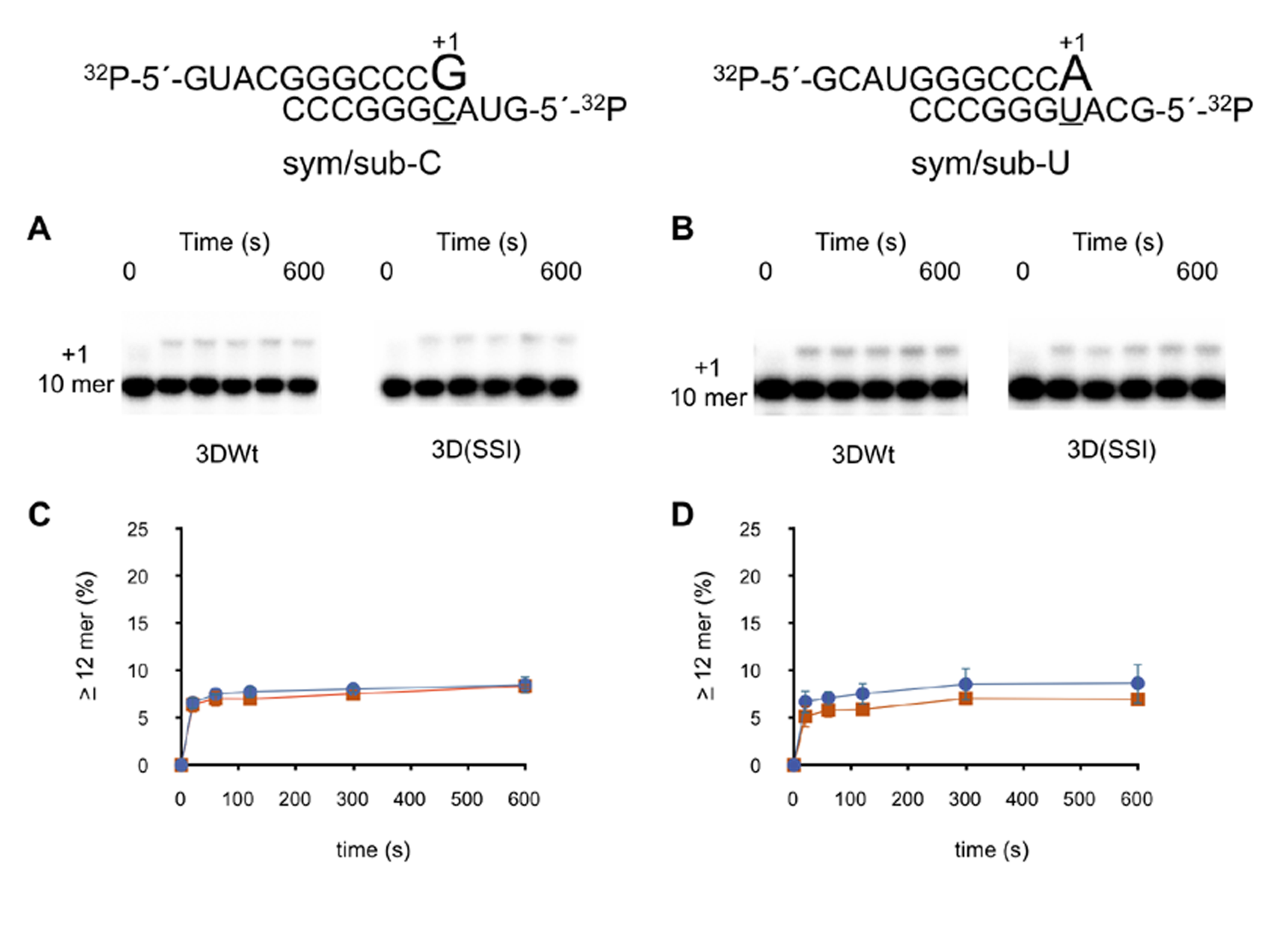

Supplement: Figure S3 — Incorporation of nucleotides into sym/sub-C and sym/sub-U by mutant FMDV polymerases. (A) Kinetics of incorporation of GMP into sym/sub-C (sequence shown at the top) by the indicated FMDV polymerases [3DWt and 3D(SSI)], performed at 37°C. The reactions were initiated by addition of 1 µM GTP after the formation of 3D-RNA complex, as described in Materials and Methods of the main text. At different time points the reaction was quenched by addition of EDTA. (B) Kinetics of incorporation of AMP into sym/sub-U (sequence shown at the top) by the indicated FMDV polymerases, performed at 37°C. The reactions were initiated by addition of 1 µM ATP after the formation of 3D-RNA complex, as described in Materials and Methods of the main text. At different time points the reaction was quenched by addition of EDTA. (C) Percentage of primer elongated to position +1 (11 mer or larger RNAs synthesized), calculated from the densitometric analysis of the electrophoreses shown in A. The results are the average of three independent experiments, and standard deviations are given. (D) Same as (C) for the incorporation of AMP at position +1 (11 mer) calculated from the densitometric analysis of the electrophoreses shown in (B). Procedures are detailed in Materials and Methods of the main text. (6.37 MB TIF) [file ppat.1001072.s003.tif]
